# A Hybrid SVD-Based Denoising and Self-Adaptive TMSST for High-Speed Train Axle Bearing Fault Detection

**DOI:** 10.3390/s21186025

**Published:** 2021-09-08

**Authors:** Feiyue Deng, Chao Liu, Yongqiang Liu, Rujiang Hao

**Affiliations:** 1 State Key Laboratory of Mechanical Behavior and System Safety of Traffic Engineering Structures, Shijiazhuang Tiedao University, Shijiazhuang 050043, China; dengfy@stdu.edu.cn (F.D.); haorj@stdu.edu.cn (R.H.); 2 School of Mechanical Engineering, Shijiazhuang Tiedao University, Shijiazhuang 050043, China; ziyou2050@163.com

**Keywords:** axle bearing, time−frequency analysis, singular value decomposition, time-reassigned synchrosqueezing transform, fault detection

## Abstract

Fault detection of axle bearings is crucial to promote the safe, efficient, and reliable running of high-speed trains. In recent decades, time−frequency analysis (TFA) techniques have been widely used in mechanical equipment fault diagnoses. Time-reassigned multisynchrosqueezing transform (TMSST), as a novel time−frequency representation (TFR) algorithm, is more suitable for dealing with strong frequency-varying signals. However, TMSST TFR results are subject to noise interference. It is difficult to extract the accurate time−frequency (TF) fault feature of the axle bearing under a complex working environment. In addition, determination of the TMSST algorithm parameters depends on the personnel’s subjective experience. Therefore, the TMSST result has a great randomicity and has the disadvantage of having a poor reliability. To address the above issues, a hybrid SVD-based denoising and self-adaptive TMSST is proposed for axle bearing fault detection in this paper. The main improvements of the proposed algorithm include the following two aspects: (1) An SVD-based denoising method using the maximum SV mean to determine the reasonable SV order is adopted to eliminate noise interference and to reserve useful fault impulse information. (2) A new evaluation metric, named time−frequency spectrum permutation entropy (TFS-PEn), is put forward for the quantitative evaluation of the performance of TFR for the TMSST, and then a water cycle algorithm (WCA)-based optimized TMSST can adaptively determine the optimal algorithm parameters. In both the simulation and experimental tests, the superiority and effectiveness of the proposed method is compared with the TMSST, short-time Fourier transform (STFT), MSST, wavelet transform (WT), and Hilbert-Huang transform (HHT) methods. The results show that the proposed algorithm has a better performance for extracting the weak fault features of axle bearing under a strong background noise environment.

## 1. Introduction

Bogies are located at both ends of high-speed train carriages and play an important role in the safe and stable operation of the trains. With the increase in train speed, the working environment of the bogies under heavy-load and long-term alternating stress conditions is becoming more and more severe. Axle bearings, as an important rotating part of the bogie, are subjected to a variety of dynamic loads and harsh working conditions during operation [1]. As a result, axle bearings are exposed to a significant risk of failure deterioration. Especially in the strong wheel-track excitation environment, the failure characteristics of axle bearings are weak and almost completely submerged in the strong noise, so the fault diagnosis of axle bearings is of great importance.

The collision of the damaged rotating roller will generate a series of periodic impacts and corresponding resonances when the surface of the bearing component fails. The fault characteristic frequencies of different types of bearing failures are varied. However, when overwhelmed by heavy noise, the fault impulse feature generated by an incipient fault is too weak to detect directly. This has led to a considerable amount of research on the vibration-based diagnosis of bearings in the last decades. Many signal processing methods, such as the spectral kurtosis (SK) algorithm [2,3], morphological filter [4], sparse representation [5], and time−frequency analysis (TFA) [6,7], have been explored over the years for bearing fault diagnosis. Among them, the TFA techniques can convert the one-dimensional (1D) time-domain signal into a two-dimensional (2D) time−frequency (TF) feature distributed along the time and frequency directions. It can effectively describe the variation of the frequency components of a non-stationary signal with time. In addition, some advanced time−frequency decomposition methods have been emerging. Chegini et al. [8] proposed an empirical wavelet transform (EWT) method for early fault detection and for diagnosing the fault pattern of bearings. Xing et al. [9] developed a bearing fault diagnosis method based on variational mode decomposition (VMD), Tsallis entropy, and the fuzzy C-means clustering algorithm. Pan et al. [10] presented a symplectic geometry mode decomposition method for rotating machinery compound fault diagnosis, which can decompose a time signal into a series of mode components. Chen et al. [11] adopted an adaptive chirp mode decomposition to extract the fast fluctuating instantaneous frequency of the signal of the rub-impact rotor, and achieved a good result.

Traditional TFA methods, such as short-time Fourier transform (STFT), Wigner−Ville distribution (WVD), and wavelet transform (WT), have been widely employed for fault signal analyses. Despite the respective good performances of these techniques, shortcomings also remain that limit their practical application. Because of the Heisenberg uncertainty principle, STFT and WT technologies are unable to obtain a high-accuracy time−frequency resolution [12]. WVD is not suitable for multi-component signal analysis because of cross-term interference [13]. Auger et al. [14] proposed a TF reassignment method that effectively improves the readability of the TF feature distribution. Daubechies et al. [15] proposed a synchrosqueezing transformation (SST) method based on TFR, further improving the readability of TFA and preserving the reversibility property, but it has a more serious ambiguity on the time−frequency representation (TFR). The degree of energy concentration of the TF distribution is an important indicator of the effectiveness of TFA methods. To overcome the problem of ambiguity in TFR, different kinds of SST techniques, such as WT-based SST, STFT-based SST, high-order SST, and time-reassigned SST (TSST), are constantly emerging to move the energy of each time−frequency point to the energy center of gravity and to generate a high energy concentrated TFR [16,17,18,19]. However, the above SST techniques exhibit a good TF energy concentration effect only when the analyzed signal has a constant instantaneous frequency or slow time-varying characteristics. In actual engineering, the fault impulses of the vibration signals for the rotating machinery key components, such as the bearings and gears, are transient, which have a very wide frequency band response. The above-mentioned methods are not applicable for a fault impact signal with violent instantaneous frequency changes.

Time-reassigned multisynchrosqueezing transform (TMSST) is the latest TFA method proposed by Yu et al. [20], which can deal with a strong frequency-varying signal and obtain a highly concentrated TFR to characterize the fault impulse. Although the TMSST method is suitable for processing transient impulse fault signals and can solve the blurry TFR problem of the traditional SST methods, there are still two deficiencies that seriously limit its application in practical engineering.

(1) The TMSST method is less robust to noise interference. The key components of the rotating machinery often work under very harsh conditions, and the measured vibration signals contain a large number of background noise interference components. The fault feature information is immersed in heavy background noise, which leads to fault impulses that are difficult to recognize. TMSST is only effective for fault impulse components of the signals, but cannot eliminate the interference of noise.
(2) The self-adaptability of the TMSST is relatively poor. In practical applications, two algorithm parameters of the TMSST need to be set in advance, and they have a significant influence on the results of the TFR. So far, the choice of the TMSST algorithm parameters still depends on human experience, which leads to a high uncertainty for the TFR results.

To tackle the above problems and expand the practical application of the TMSST, a framework combining singular value decomposition (SVD)-based denoising and self-adaptive TMSST is presented in this paper. The proposed method can eliminate noise interference as much as possible, and can effectively extract weak fault impulse features for axle bearing fault diagnosis. The main contributions of the work proposed in this paper are summarized as follows.

(1) An SVD-based denoising method is introduced to eliminate noise interference. The SVD technique is reviewed as a competitive noise reduction method, which has been widely used in signal denoising [21,22]. Hence, for the problem that the reconstruction singular value (SV) order is difficult to determine in SVD, a maximum SV mean method is proposed in this paper to implement the self-adaptive determination of the SV order. The useful fault impulse components of the signal are retained and the noise components are removed after SV reconstruction.
(2) Adaptive optimization TMSST is developed to acquire the optimal algorithm parameters and extract the TF fault feature information. A new evaluation metric, time−frequency spectrum permutation entropy (TFS-PEn), is proposed to quantitatively evaluate the TFR performance of the TMSST. To further improve the adaptability of TMSST, an optimized water cycle algorithm (WCA) is introduced to determine the algorithm parameters adaptively.

The rest of the paper is structured as follows. SVD-based denoising based on the maximum SV mean is described in Section 2. The TMSST method is briefly described in Section 3. The proposed method is detailed in Section 4. Numerical and experimental examples are illustrated in Section 5. Conclusions are drawn in Section 6.

## 2. SVD-Based Denoising Theory

The SVD-based denoising algorithm has two remarkable advantages. (1) SVD is a non-parametric technique and is easy to implement. For a given matrix, it can be decomposed into two orthogonal matrices and some corresponding singular values without any parameters. Some denoising algorithms, such as the Kalman filter algorithm and wavelet transform filter algorithm, need to set relevant parameters in advance. (2) The essence of SVD is to retain the useful information in the signal subspace and to remove the interference components in the noise subspace. So, the SVD-based denoising algorithm does not introduce additional components into the denoising process. It does not pollute the raw signal, which is different from other denoising algorithms, such as morphology filter and stochastic resonance denoising. Based on the above analysis, the SVD-based denoising algorithm is suitable for signal denoising preprocessing.

### 2.1. SVD

Assuming that $X=\{x_{1},x_{2},...,x_{N}\}$ is a 1D vibration signal, the Hankel matrix of *X* is formed as

(1) $$A_{m\times n}=\left(\begin{array}{cccc} x(1) & x(2) & \cdots & x(n)\\ x(2) & x(3) & \cdots & x(n+1)\\ \vdots & \vdots & \vdots & \vdots \\ x(m) & x(m+1) & \cdots & x(N) \end{array}\right)$$

where 1 < *m* < *N*, *n* = *N* − *m* + 1. *m* is the rows of the matrix and *n* is the columns of the matrix. In order to enhance the performance of the SVD, the Hankel matrix should be decomposed as fully as possible [23]. When constructing the Hankel matrix, the number of rows and columns of the matrix should be as equal as possible according to the length of the analyzed signal. In the course of practice, n=N/2 when N is an even number and n=(N+1)/2 when N is an odd number.

For matrix $\;A_{m\times n}$, the following mathematical formula can be obtained according to the theory of the SVD.

(2) $$A_{m\times n}=U_{m\times l}\Lambda V_{n\times l}^{T}$$

where $U_{m\times l},V_{n\times l}^{T}$ are the orthogonal matrixes and Λ is a diagonal matrix with the size of  l×l. The diagonal elements are

(3) $$\Lambda =diag(\sigma_{1},\sigma_{2},...,\sigma_{k})$$

where $\sigma_{1}\geq \sigma_{2}\geq \cdots \geq \sigma_{k}\geq 0$, k=min(m,n). $\sigma_{1},\sigma_{2},...,\sigma_{k}$ are the SVs of the matrix $\;A_{m\times n}$. For a bearing vibration signal with noise, the matrix $A_{m\times n}$ can be considered as follows:

(4) $$A_{m\times n}=D_{m\times n}+W_{m\times n}$$

where $D_{m\times n}$ belongs to the fault signal space and $W_{m\times n}$ belongs to the noise signal space. This reveals that the SVs that belong to the fault useful signal and noise signal have different distribution features. The useful signal can be obtained through reconstruction with some SVs that represent the fault impulse components. 

### 2.2. Signal Reconstruction

**Figure 1:**
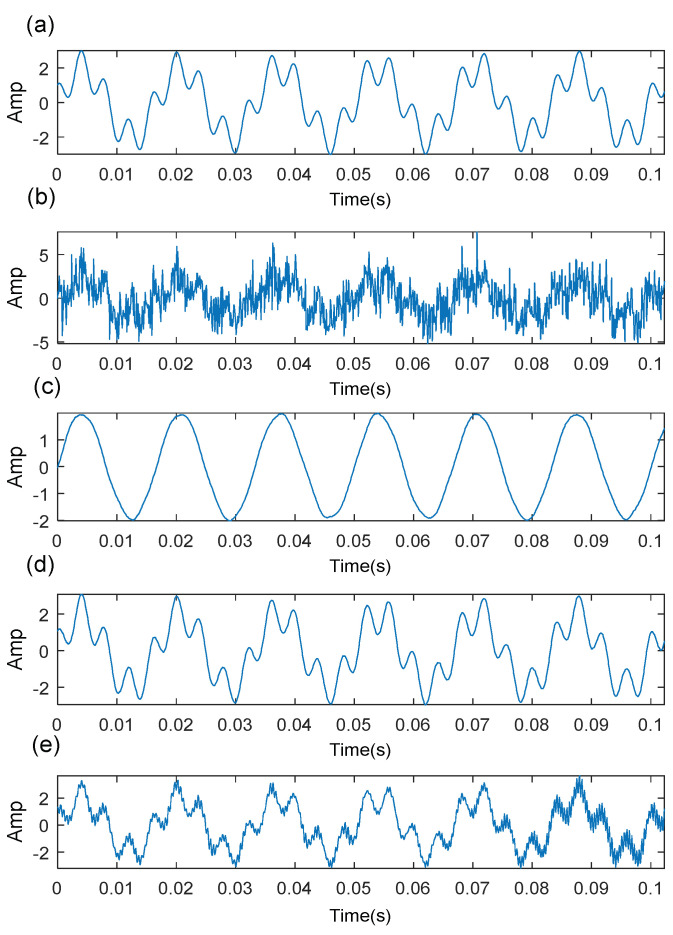
SVD-based denoising based on different SVorders: (**a**) original signal; (**b**) noisy signal; (**c**) constructed signal based on order 2; (**d**) constructed signal based on order 4; (**e**) constructed signal based on order 8.

The performance of SVD-based denoising depends on the choice of the SV order. Figure 1a shows a simulated signal. When the simulated signal contains a strong noise, it is difficult to identify the original signal waveform (Figure 1b). Figure 1c–e shows the reconstructed signal based on different SV orders. The useful components of the signal cannot be extracted completely if the SV order is not enough, while the noise components cannot be eliminated effectively if the SV order is over the upper limit. 

Through the above analysis, knowing how to make a reasonable choice for the SV order is crucial for SVD-based denoising. The Hankel matrix, described by Equation (1), shows that two adjacent row vectors lag only one data point. Hence, adjacent row vectors are highly similar sequences with a strong resistance. The Hankel matrix consists of useful signal components and has a significant feature that the SVs are larger in the first few values and the rest are much smaller. However, the difference in SVs for a noise matrix is similar because the two adjacent row vectors in the noise matrix have a bad correlation. Based on these analyses, the maximum SV mean method is proposed in order to determine the reasonable SV order in this paper. The SV mean is illustrated with the following equation

(5) $$Z_{i}=\frac{\sigma_{i-1}-\sigma_{i+1}}{2}$$

where $Z_{i}$ is the *i*-th SV mean. $k=\arg(\max Z_{i})$ is the optimal SV order. The appearance of the maximum SV mean is caused by the irrelevance of the fault component and the noise interference component in the signal; therefore, the maximum mean value can be seen as a dividing line between the useful signal and the noise signal. Only the first *k* SVs are reserved for matrix reconstruction to reconstruct the denoising signal. 

## 3. Time-Reassigned Multisynchrosqueezing Transform (TMSST)

### 3.1. Time-Reassigned Synchrosqueezing Transform(TSST)

A mono-component signal can be expressed as follows:

(6) $$\hat{s}(w)=A(\omega )e^{i\varphi (\omega )}$$

where A(ω) and φ(ω) represent the amplitude and phase of the signal, respectively. $\hat{s}(w)$ is transformed into the TF domain by STFT using a moving window function $\hat{g}(\xi )$

(7) $$G(t,\omega )={\left(2\pi \right)}^{-1}\int_{-\infty }^{+\infty }\hat{s}(\xi )\hat{g}(\xi -w)e^{i(\xi -\omega )t}d\xi$$

The first-order Taylor expansion of $\hat{s}(w)$ is written as

(8) $$\hat{s}(\xi )=A(w)e^{i(\varphi (w)+\varphi^{\prime }(w)(\xi -\omega ))}$$

Substituting Equation (8) into Equation (7) results in the following

(9) $$\begin{array}{cc} G(t,\omega ) & =(2\pi {)}^{-1}\int_{-\infty }^{+\infty }A(\omega )e^{i(\varphi (\omega )+\varphi^{'}(\omega )(\xi -\omega ))}\overset{⌢}{g}(\xi -\omega )e^{i(\xi -\omega )t}d\xi \\ & =A(\omega )e^{i\varphi (\omega )}g(t+\varphi^{'}(\omega )) \end{array}$$

where φ'(ω) represents the group delay (GD). It is observed that the TF energy of $\hat{s}(\omega )$ spreads around the GD trajectory in Equation (9). To enhance the TF energy concentration, TSST is proposed to derive a 2D GD estimate as follows:

(10) t^(t,ω)=Re(i∂ωG(t,ω)G(t,ω))

where Re() is the real part. Substituting Equation (9) into Equation (10) results in the following:

(11) $$\hat{t}(t,\omega )=-\varphi^{'}(\omega )$$

The blurry TF energy is squeezed into the GD trajectory through the operation of a 1D integration along the time direction.

(12) $$Ts(u,\omega )=\int_{-\infty }^{+\infty }G(t,\omega )\delta (u-\hat{t}(t,\omega ))dt$$

Combining Equations (10), (12), and (13), we obtain the following:

(13) $$\begin{array}{cc} Ts(u,\omega ) & =(2\pi {)}^{-1}\int_{-\infty }^{+\infty }\int_{-\infty }^{+\infty }\hat{s}(\xi )\overset{⌢}{g}(\xi -\omega )e^{i(\xi -w)t}\delta (u+\varphi^{'}(\omega ))d\xi dt\\ & =\hat{s}(\xi )\overset{⌢}{g}(0)\delta (u+\varphi^{'}(\omega )) \end{array}$$

It can be seen from Equation (13) that the blurry TF energy of $\hat{s}(w)$ can be squeezed into the GD trajectory in the TSST. However, it only achieves a good result for a signal with weak frequency-varying characteristics. 

### 3.2. Time-Reassigned Multisynchrosqueezing Transform (TMSST)

The second-order Taylor expansion of $\hat{s}(\omega )$ is obtained

(14) $$\hat{s}(\xi )=A(\omega )e^{i(\varphi (\omega )+\varphi^{'}(\omega )(\xi -\omega )+0.5\varphi^{''}(\omega ){\left(\xi -\omega \right)}^{2})}$$

The Gaussian window function used in STFT is

(15) $$g(\omega )=\sqrt{2\sigma \pi }e^{-0.5\sigma \omega^{2}}$$

Substituting Equations (14) and (15) into Equation (7), we obtain the following:

(16) $$\begin{array}{cc} G(t,\omega ) & =(2\pi {)}^{-1}\int_{-\infty }^{+\infty }A(\omega )e^{i(\varphi (\omega )+\varphi^{'}(\omega )(\xi -\omega )+0.5\varphi^{''}(\omega ){\left(\xi -\omega \right)}^{2})}\sqrt{2\sigma \pi }e^{-0.5\sigma {\left(\xi -\omega \right)}^{2}}e^{i(\xi -\omega )t}d\xi \\ & =A(\omega )e^{i\varphi (\omega )}\sqrt{\frac{\sigma }{\sigma -i\varphi^{''}(\omega )}}e^{-\frac{{\left(t+\varphi^{'}(\omega )\right)}^{2}}{2\sigma -2i\varphi^{''}(\omega )}} \end{array}$$

The 2D GD estimate of Equation (16) can be obtained as follows

(17) $$\hat{t}(t,\omega )=-\varphi^{\prime }(\omega )+\frac{\varphi^{\prime \prime }{\left(\omega \right)}^{2}}{\sigma^{2}+\varphi^{\prime \prime }{\left(\omega \right)}^{2}}\left(t+\varphi^{\prime }(\omega )\right)$$

Now, substituting $t=-\varphi^{'}(\omega )$ into Equation (17), we obtain the following

(18) $$\hat{t}(-\varphi^{'}(\omega ),\omega )=-\varphi^{'}(\omega )$$

The fixed-point iterative algorithm of $-\varphi^{'}(\omega )$ is used to reduce the error $-\varphi^{'}(\omega )$ and $\overset{⌢}{t}(t,w)$. The iteration results from the 1-th to *N*-th are

(19) $$\left\{\begin{array}{ccc} \; & \hat{t}\left(\hat{t}\left(t,\omega \right),\omega \right)=-\varphi^{{}^{\prime }}\left(\omega \right)+{\left(\frac{{\varphi^{{}^{\prime }}}^{{}^{\prime }}{\left(\omega \right)}^{2}}{\sigma^{2}+\varphi^{{}^{\prime \prime }}{\left(\omega \right)}^{2}}\right)}^{2}\left(t+\varphi^{{}^{\prime }}\left(\omega \right)\right)\\ \; & \hat{t}\left(\hat{t}\left(\hat{t}\left(t,\omega \right),\omega \right),\omega \right)=-\varphi^{{}^{\prime }}\left(\omega \right)+{\left(\frac{{\varphi^{{}^{\prime }}}^{{}^{\prime }}{\left(\omega \right)}^{2}}{\sigma^{2}+\varphi^{{}^{\prime \prime }}{\left(\omega \right)}^{2}}\right)}^{3}\left(t+\varphi^{{}^{\prime }}\left(\omega \right)\right)\\ \; & \cdots & \\ \; & {\hat{t}}^{\left[N\right]}\left(t,\omega \right)=-\varphi^{{}^{\prime }}\left(\omega \right)+{\left(\frac{{\varphi^{{}^{\prime }}}^{{}^{\prime }}{\left(\omega \right)}^{2}}{\sigma^{2}+\varphi^{{}^{\prime \prime }}{\left(\omega \right)}^{2}}\right)}^{N}\left(t+\varphi^{{}^{\prime }}\left(\omega \right)\right) \end{array}\right.$$

When the iteration number *N* reaches infinity, the following result can be obtained:

(20) $$\underset{N\to \infty }{\lim}{\hat{t}}^{\left[N\right]}(t,\omega )=-\varphi^{'}(\omega )$$

So, Equation (13) can be written as

(21) $$Ts^{\left[N\right]}(u,\omega )=\int_{-\infty }^{+\infty }G(t,\omega )\delta (\zeta -{\hat{t}}^{\left[N\right]}(t,\omega ))dt$$

After a sufficient number of iterations, we obtain

(22) $$\underset{N\to \infty }{\lim}Ts^{\left[N\right]}(u,\omega )=\hat{s}(\omega )\overset{⌢}{g}(0)\delta (u+\varphi^{'}(\omega ))$$

The TMSST method can squeeze the TF energy of a strong frequency-varying signal into the GD trajectory through multiple fixed-point iterations. In the TMSST result, there are a series of significant TF amplitude points that denote the fault impulse interval. The maximum value of the TF envelope spectrum (TFES) for each amplitude point can be used to represent the fault impulse feature, which is calculated as follows

(23) $$TFES(\omega )=\max\left|\int_{-\infty }^{+\infty }(\left|Ts^{\left[N\right]}(t,\omega )\right|-\phi (\omega ))e^{-i\xi t}dt\right|$$

where ϕ(ω) is the mean value at the frequency point ω in the TMSST result. The maximum TFES values can be applied to extract the periodic fault impulse feature.

**Figure 2:**
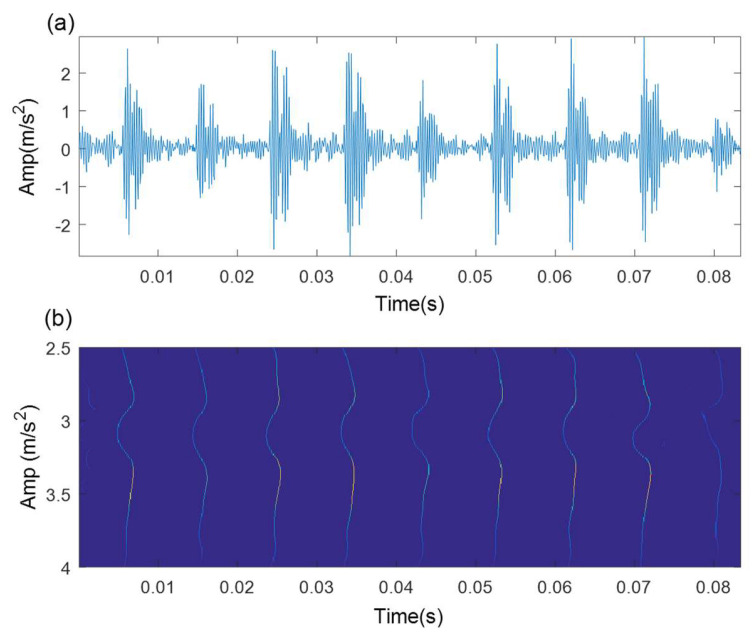
TMSST result of a simulated signal: (**a**) time-domain waveform; (**b**) TMSST result.

**Figure 3:**
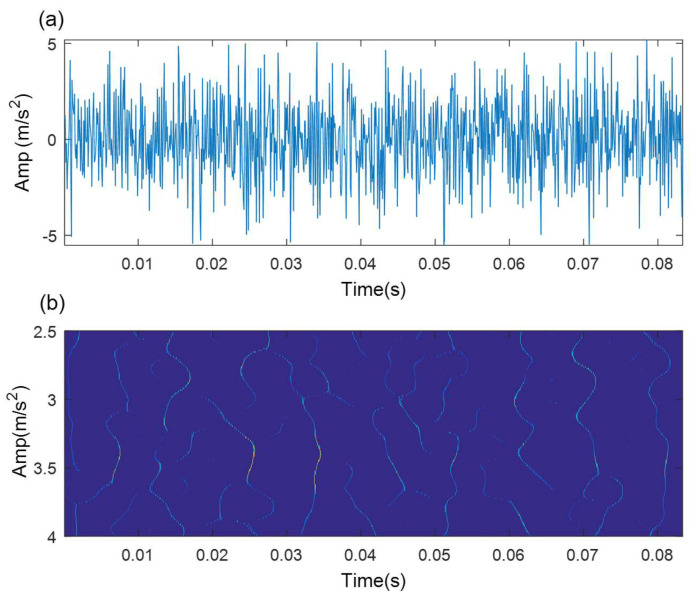
TMSST result of a simulated signal with noise: (**a**) time-domain waveform; (**b**) TMSST result.

The antinoise capability of TMSST is poor because the noise components cannot be squeezed into the GD trajectory through the TMSST. Figure 2a gives a simulated fault impulse signal without any noise, and Figure 2b shows its TMSST result. It is found that every TF energy trajectory can represent the corresponding fault impulse in the simulated signal. The simulated signal adding SNR = −5dB Gaussian white noise and its TMSST result are illustrated in Figure 3a,b, respectively. The TF energy trajectories are blurred and confusing, and do not represent the fault characteristics. 

## 4. The Proposed Method

### 4.1. Time−Frequency Spectrum Permutation Entropy (TFS-PEn)

**Figure 4:**
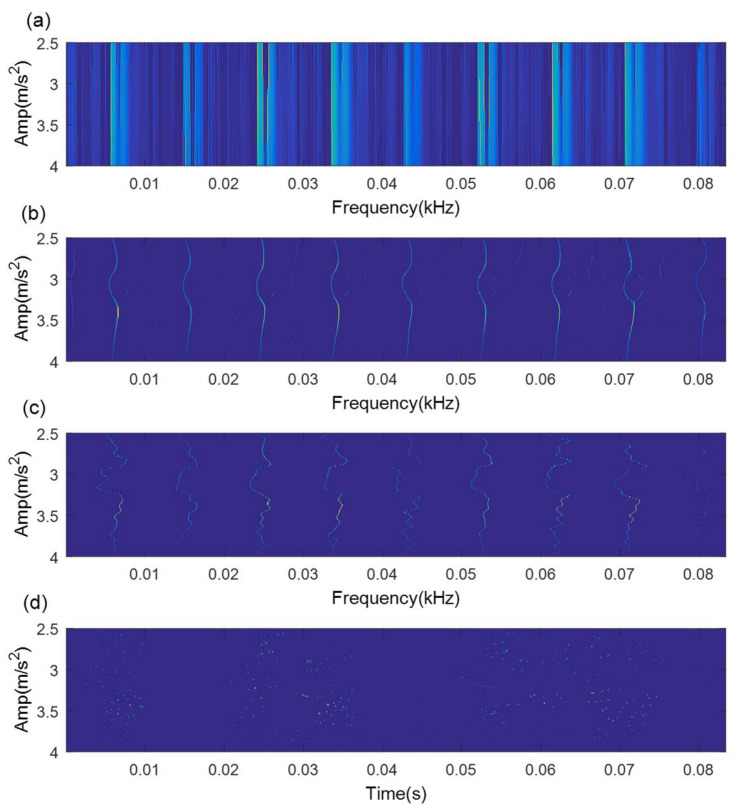
TMSST result of a simulated signal based on different $h_{w}$ values: (**a**) $h_{w}=10$; (**b**)$h_{w}=100$; (**c**) $h_{w}=300$; (**d**)$h_{w}=500$.

In the course of TMSST implementation, there are two algorithm parameters (moving window width $h_{w}$ and number of iterations $h_{num}$) that need to be confirmed in advance. Figure 4 illustrates the TMSST results of the simulated signal shown in Figure 2a with different $h_{w}$ values. As illustrated in Figure 4, a small $h_{w}$ value causes a bad TF energy concentration, while a large $h_{w}$ value leads to a blurry TF energy representation. Therefore, the selection of algorithm parameters for the TMSST has an important effect on its TFR performance. To avoid the blindness of the parameter selection and to improve the robustness and reliability of the traditional TMSST, a new metric called TFS-PEn is proposed to quantitatively evaluate the TFR performance of the TMSST.

According to Equation (22), the TMSST result is integrated along the frequency axis to acquire the time−frequency spectrum (TFS) as follows

(24) $$E_{Ts}=\int_{-\infty }^{+\infty }\hat{s}(\omega )\overset{⌢}{g}(0)\delta (u+\varphi^{'}(\omega ))d\omega$$

TFS represents the distribution of the TF energy along the time axis in the TMSST result. When a highly concentrated TFR of a fault impulse appears in the TMSST result, the TFS shows an obvious regularity feature. Alternatively, if the TFR of the TMSST result is blurry, the distribution of the TFS has a large randomness. To further estimate the TFES result, permutation entropy (PEn) is introduced to calculate the TFS-PEn. 

PEn is a parameter of average entropy that was first proposed by Bandt et al. [24]. It can be utilized to describe the complexity of a time series or a measured signal in a physical system reconstructed by the phase space. In addition, PEn also takes into account the nonlinear behavior characteristic of the signal sequence, which is very suitable for analyzing the fault vibration signals of the bearing [25]. For the given TFS series $\left\{E_{Ts}(i),i=1,2,\cdots ,N\right\}$ of size *N*, an embedding procedure is used to form a vector $X_{E_{Ts}}=[E_{Ts}(i),E_{Ts}(i+\tau ),\cdots ,E_{Ts}(i+(m-1)\tau )]$ arranged in an increasing order, where the embedding dimension is *m* and the lag is τ. When *m* is fixed, there are m!=1×2×⋯×N possible permutations. For a given permutation π, f(π) represents its frequency in the TFES series. Assuming the relative frequency is p(π)=f(π)/(N−(m−1)τ), the PEn for the TFS series is written as follows:

(25) $$H_{p}(m)=-\sum_{m=1}^{m!}p(\pi )\ln p(\pi )$$

The corresponding normalized entropy is given as follows:

(26) $$H_{p}=H_{p}(m)/\ln(m!)$$

In the calculation of PEn, the embedded dimension and the lag are set to *m* = 6 and τ=3, separately, according to [26].

**Figure 5:**
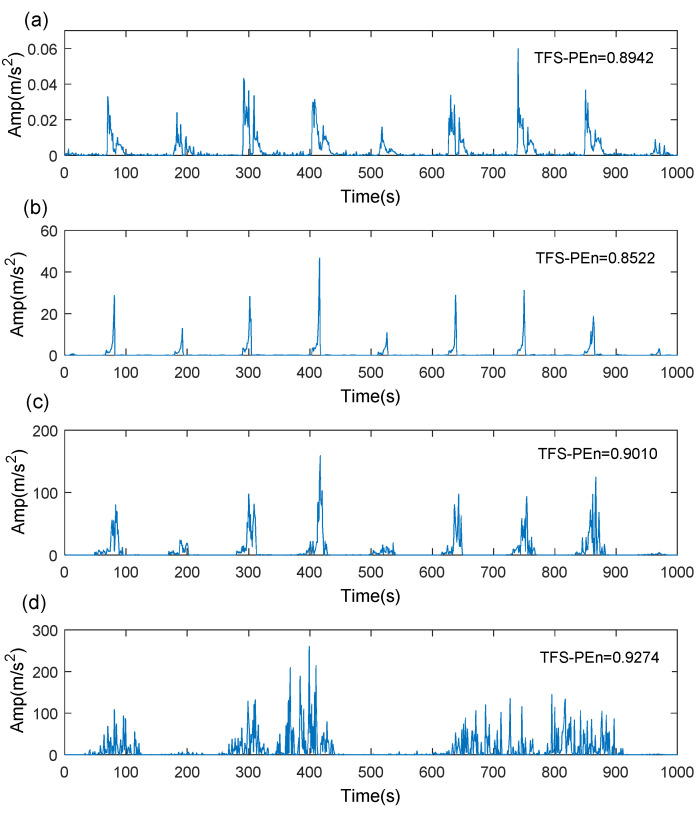
TFS results and their TFS-PEns for a simulated signal based on different $h_{w}$ values: (**a**) $h_{w}=10$; (**b**) $h_{w}=100$; (**c**) $h_{w}=300$; (**d**) $h_{w}=500$.

When the TFS series shows regular fault impulse characteristics, the TFS-PEn value is smaller and, vice versa, TFS-PEn value is larger. TMSST results with different $h_{w}$ values shown in Figure 4 are used as an example to measure the TFS-PEn value. The corresponding TFS results and their TFS-PEns are displayed in Figure 5. It can be clearly observed that the TFS series based on $h_{w}=100$ exhibits a more obvious regular fault impulse feature with a minimum TFS-PEn value compared with the other TFS series. The reduction in the periodic fault components leads to an increase in the complexity of the TFS series and to an increase in the TFS-PEn value. Therefore, the minimum TFS-PEn evaluation criterion is used to optimize the selection of the algorithm parameters in the TMSST method.

### 4.2. Water Cycle Algorithm (WCA)-Based Optimized TMSST

**Figure 6:**
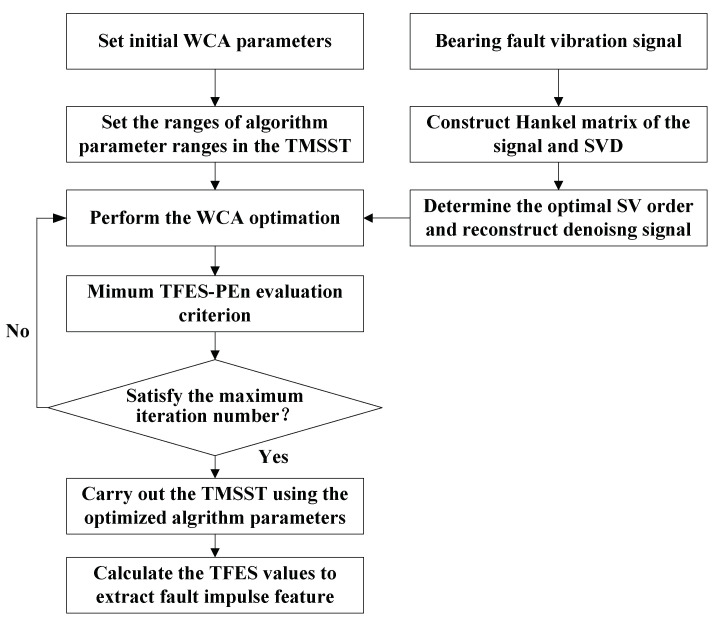
Flowchart of the proposed method.

To further improve the efficiency and accuracy of the algorithm parameter identification, a water cycle algorithm (WCA) is introduced into the TMSST to adaptively acquire the optimal parameters in this paper. As a novel metaheuristic optimization algorithm, WCA is used for solving the constrained engineering optimization problems, which has many advantages, such as fast convergence, excellent capability for optimal weighting searching, high accuracy, and good robustness [27]. For details of the WCA implementation, please refer to [28]. The flowchart of the proposed method is shown in Figure 6, and its specific steps are as follows:

(1) Construct the Hankel matrix of the analyzed signal according to Equation (1) and perform the SVD on the Hankel matrix.
(2) Acquire the SVs of the Hankel matrix and determine the optimal SV order according to the maximum SV mean method, and then reconstruct the denoising signal according to the optimal SV order.
(3) Set the WCA’s initial parameters. The number of rivers and sea is $N_{sr}=5$, the total number of population is $N_{pop}=15$, the evaporation condition constant is $d_{\max}=1\times 10^{-5}$, and the maximum number of iteration is max_it=50. In the TMSST implementation, the ranges of parameters $h_{w}$ and $h_{num}$ are set to [1, 500] and [1, 20], respectively.
(4) The minimum TFS-PEn evaluation criterion is used in the WCA to adaptively select the optimal algorithm parameters of the TMSST. Perform the WCA optimization until the maximum iteration number is satisfied, and obtain the optimal TMSST parameters.
(5) Carry out the TMSST and calculate the maximum TFES values to extract the fault impulse features of the faulty bearing.

## 5. Simulation Study

**Figure 7:**
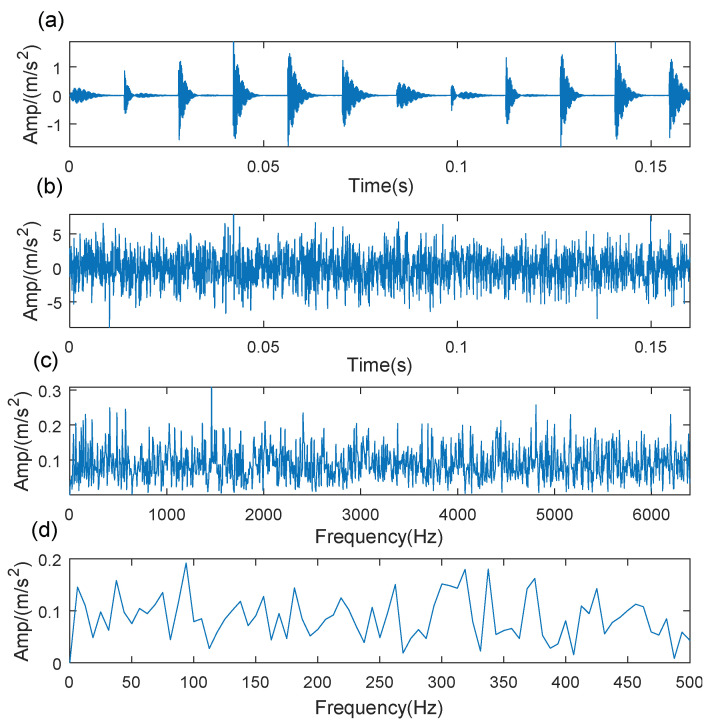
The simulated signal: (**a**) time-domain waveform of the pure signal; (**b**) time-domain waveform of the noise signal; (**c**) frequency spectrum of the noise signal; (**d**) direct envelope spectrum of the noise signal.

A simulated bearing fault signal consisting of fault impulses and noise interferences is given as follows [29]

(27) $$\left\{\begin{array}{l} h(t)=\exp(-\zeta t)\cos(2\pi f_{n}t)\\ x(t)=\sum_{i}A_{i}h(t-iT-\tau_{i})\sin(2\pi f_{r}t+\theta )+r(t) \end{array}\right.$$

where the resonance frequency is $f_{n}=4000$, damping coefficient is ζ=500, average period of fault impulse component is *T* = 14 ms, and $A_{i}=2$ is the amplitude. $\tau_{i}$ is a small fluctuation variable of the *i*-th fault impact and τ~N(0,0.05T). The sampling frequency of the simulated signal is 12,800 Hz ad the period of fault impulse is set as 14ms. In the simulated signal, *r*(*t*) is the Gaussian white noise with a signal to noise ratio (SNR = −7dB), which is white noise with a mean of 0 and standard deviation. The pure signal and mixed signal with noise are shown in Figure 7a,b, respectively. The frequency spectrum of the simulated signal is shown in Figure 7c, and the direct envelope spectrum is shown in Figure 7d. Because of the noise interference, it is difficult to identify the fault impulse components in the noise signal waveform, and the fault characteristic frequency cannot be extracted in the direct envelope spectrum either.

**Figure 8:**
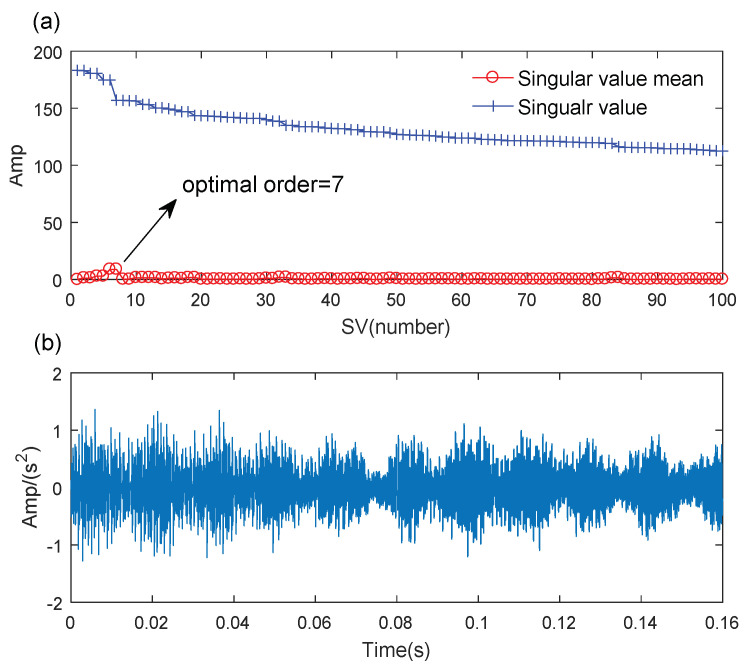
The results of the SVD for the simulated signal: (**a**) some SVs and their means; (**b**) the reconstructed signal.

**Figure 9:**
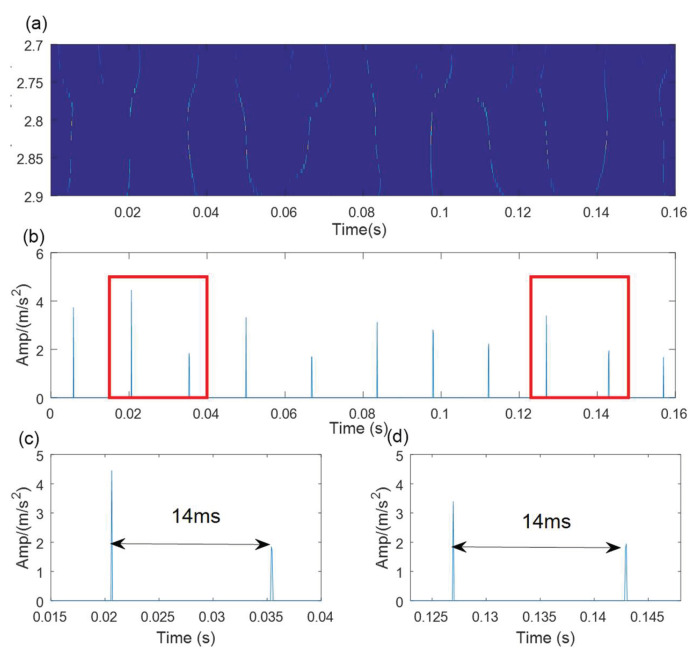
The results of the proposed method for the simulated signal: (**a**) TMSST result; (**b**) TFES result of the TMSST; (**c**,**d**) zoomed versions.

First, the SVD-based denoising method is used to process the simulated signal, and the results are shown in Figure 8. The optimal SV order is 7 according to the position of the maximum SV mean. The denoising signal is processed by the WCA-optimized TMSST, and the optimal algorithm parameters are $h_{w}=387$ and $h_{num}=11$. The TMSST result is shown in Figure 9a. A series of highly concentrated TF trajectories can be found in the TMSST result. The maximum TFES values are shown in Figure 9b, and it can be seen that the time intervals are very clear and have a regular periodic pattern. From the zoomed versions of the TMSST result shown in Figure 9c,d, it can be observed that the time intervals are equal to the pre-set fault characteristic period of the simulated signal.

## 6. Experiment Study

### 6.1. Experiment Description

**Figure 10:**
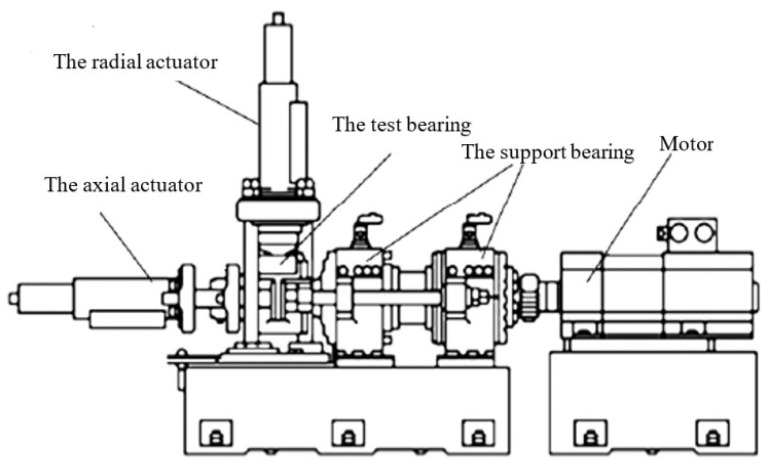
The structure of the test rig.

The fault signals of the bearings are acquired from a high-speed train axle bearing test rig. The structure of the test rig is shown in Figure 10. One end of the test rig is a support normal bearing that joins a motor, and the other end is the test axle bearing. A radial actuator and an axial actuator are mounted on the test bearing. The hydraulic loading device can apply radial and axial static loading to the test bearing. Some real track spectrums can be input into the control system to simulate the change of the real excitation of the axle bearing. Therefore, the test rig can simulate the actual situation of the axle bearing when the high-speed train is running, and can meet the operation test under different speed grades and load conditions.

**Figure 11:**
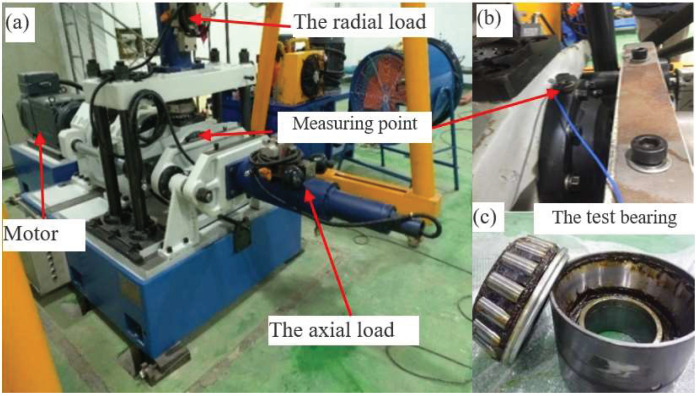
The main parts of the test rig: (**a**) axle bearing test rig; (**b**) accelerometer installation position; (**c**) faulty axle bearing.

**Figure 12:**
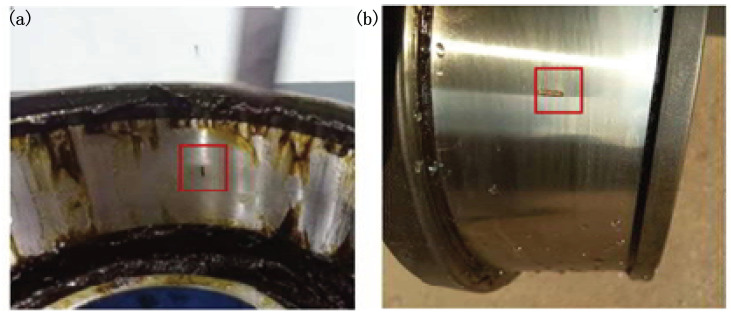
Faulty axle bearing: (**a**) outer race fault; (**b**) inner race fault.

The main parts of the test rig are illustrated in Figure 11. Detailed information of the test axle bearing is given in Table 1. The inner race and outer race surface of the bearing are processed by wire cutting to generate a small dent defect. They are shown in Figure 12. An accelerometer is mounted on the test bearing. The vibration signals are picked up with a sampling frequency 51,200 Hz. The shaft rotational speed is set to 1500 rpm (rotating frequency $f_{r}=7.75$ Hz) in the bearing inner race fault test, and the shaft speed is 1800 rpm (rotating frequency $f_{r}=7.75$ Hz) in the outer fault test. The fault impulse intervals of the outer race fault and inner race fault are 4.56 ms and 4.11 ms, respectively.

### 6.2. Fault Analysis

**Figure 13:**
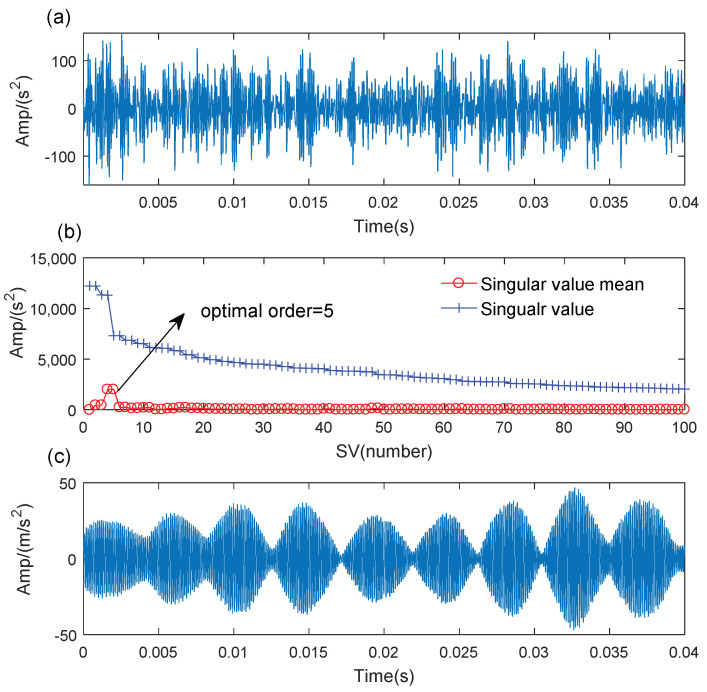
The SVD-based denoising of the outer race fault signal: (**a**) original signal; (**b**) some SVs and their means; (**c**) denoising result.

**Figure 14:**
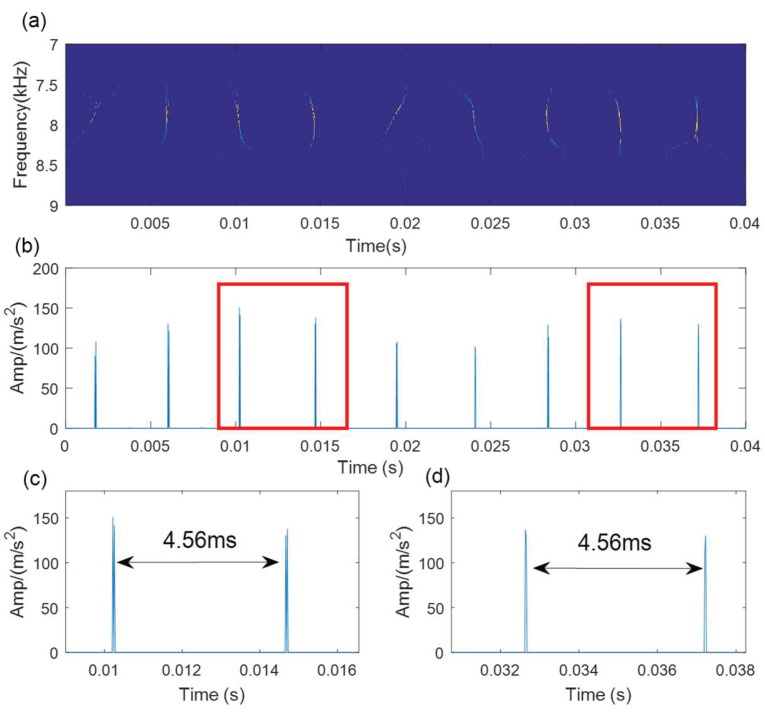
The results of the proposed method for outer race fault. (**a**) MTSST result; (**b**)TFES result of the TMSST; (**c**,**d**) zoomed versions.

Figure 13a shows the time-domain waveform of the bearing outer race fault signal. It is difficult to find any evident fault impulse components because of the noise interference. The SVD-based denoising results are illustrated in Figure 13. The optimal SV order is 5 according to the maximum SV mean in Figure 13b. The denoising signal is processed by the WCA-optimized TMSST, and the optimal TMSST parameters are $h_{w}=344$ and $h_{num}=11$. Some clear TF trajectories can be found in the TMSST result shown in Figure 14a, and they present a clear periodic pattern. The maximum TFES values are calculated as shown in Figure 14b. The zoomed versions of the TFES result illustrated in Figure 14c,d demonstrate that the impulse interval is equal to the fault cycle interval. It can be concluded that the proposed method is feasible for bearing fault diagnosis.

**Figure 15:**
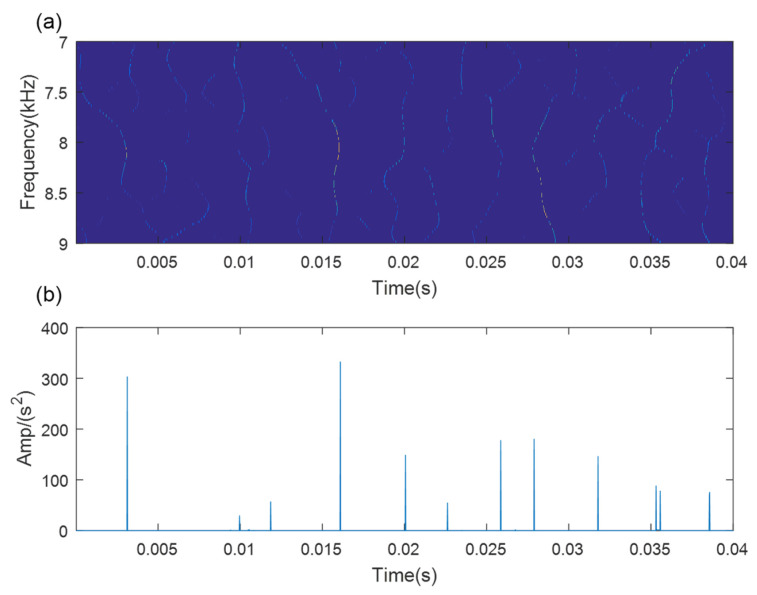
The results of the traditional TMSST method for the outer fault signal: (**a**) MTSST result; (**b**) TFES result of the TMSST.

As a comparative analysis, the traditional TMSST based on the optimal algorithm parameters is directly used for the analysis of the bearing outer race fault signal. The TMSST result is displayed in Figure 15a. It can be seen that the TF trajectories are blurred and muddled. We cannot extract any obvious TF fault features in the TMSST result. The maximum TFES values are shown in Figure 15b. The impulse intervals are not equal to each other. The results show that the traditional TMSST method failed to detect the fault feature due to the strong noise interference. Therefore, the SVD-based denoising method is necessary and useful.

**Figure 16:**
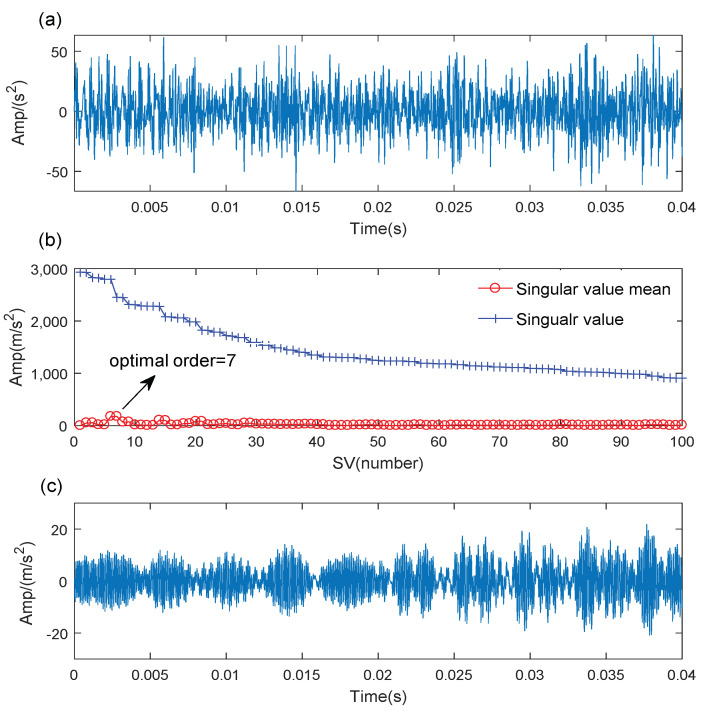
The SVD-based denoising of the inner race fault signal: (**a**) original signal; (**b**) some SVs and their means; (**c**) denoising result.

**Figure 17:**
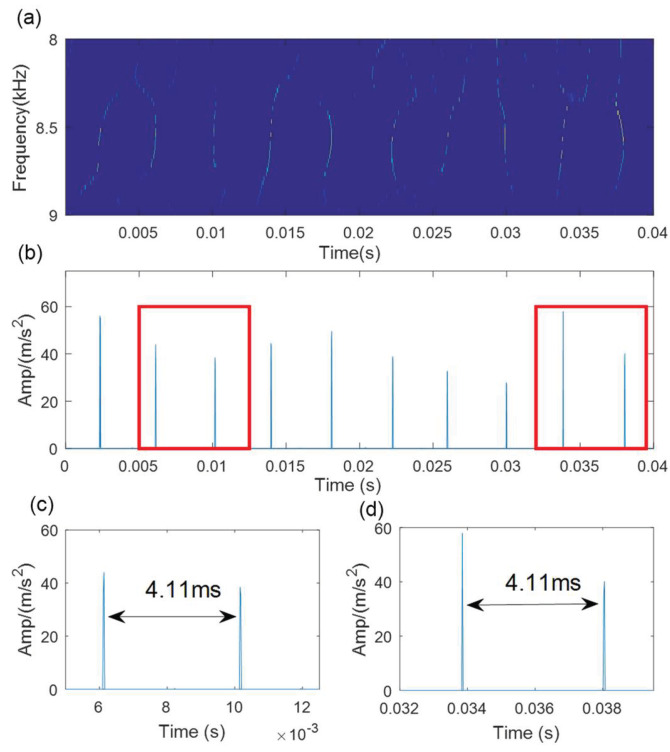
The results of the proposed method for the inner race fault: (**a**) MTSST result; (**b**) TFES result of the TMSST; (**c**,**d**) zoomed versions.

The time-domain waveform of the axle bearing inner fault signal is shown in Figure 16a. The optimal SV order is chosen as 7 according to the maximum SV mean in Figure 16b, and the denoising result is given in Figure 16c. The results of the WCA-optimized are $h_{w}=423$ and $h_{num}=6$. The TMSST result is displayed in Figure 17a, and the TF trajectories exhibits a good TFR performance. The inner race fault impulse can be accurately extracted in Figure 17b,c.

**Figure 18:**
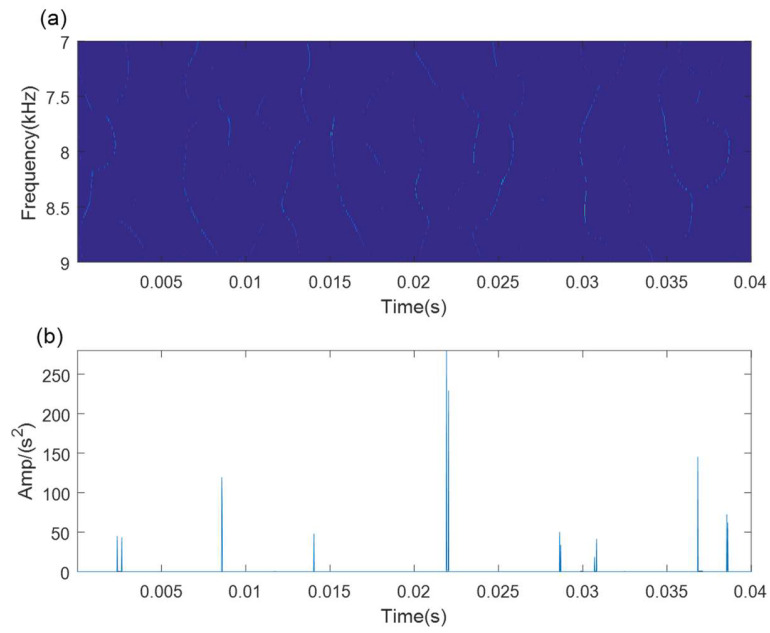
The analysis results of the direct TMSST method for the inner fault signal:(**a**) MTSST result; (**b**) TFES result of the TMSST.

The result of the traditional TMSST using the same algorithm parameters is given in Figure 18a. The TF trajectories are blurred, and the fault impulses cannot be identified accurately. Figure 18b shows that the time intervals of the maximum TFES values vary from each other. It failed to extract fault features using the traditional TMSST method.

### 6.3. Comparison Analysis

**Figure 19:**
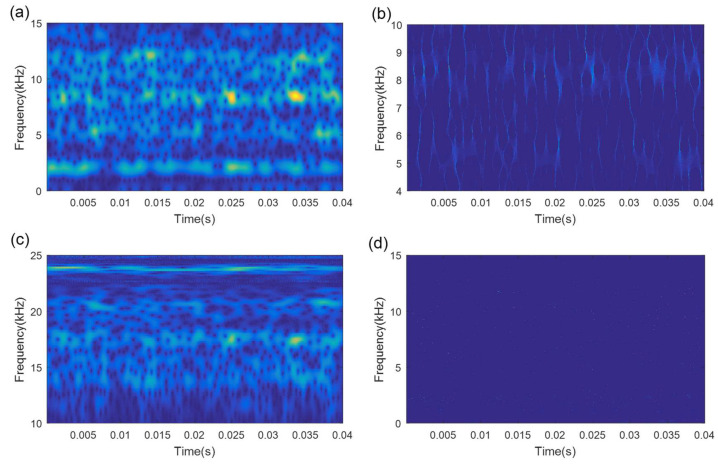
The analysis results of the compared methods for the initial outer race fault signal: (**a**) STFT result; (**b**) MSST result; (**c**) WT result; (**d**) HHT result.

**Figure 20:**
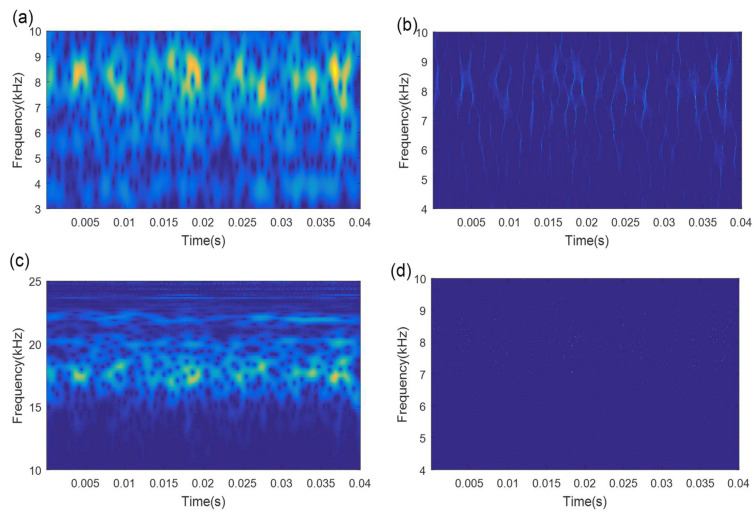
The analysis results of the compared methods for the initial inner race fault signal: (**a**) STFT result; (**b**) MSST result; (**c**) WT result; (**d**) HHT result.

In order to demonstrate the superiority and effectiveness of the proposed method, four existing common TFA methods, STFT, MSST, WT, and Hilbert-Huang transform (HHT), are utilized to analyze the axle bearing fault signals. Figure 19 and Figure 20 display the analysis results of the above comparison methods for the initial signal without denoising. The results show that the TFRs of all four methods are very blurred and the outer race and inner race fault features cannot be identified accurately.

**Figure 21:**
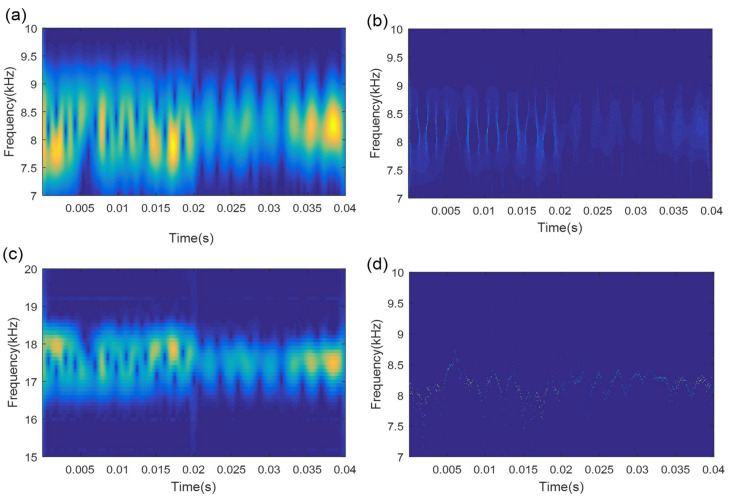
The analysis results of the compared methods for the outer race fault denoised signal: (**a**) STFT result; (**b**) MSST result; (**c**) WT result; (**d**) HHT result.

**Figure 22:**
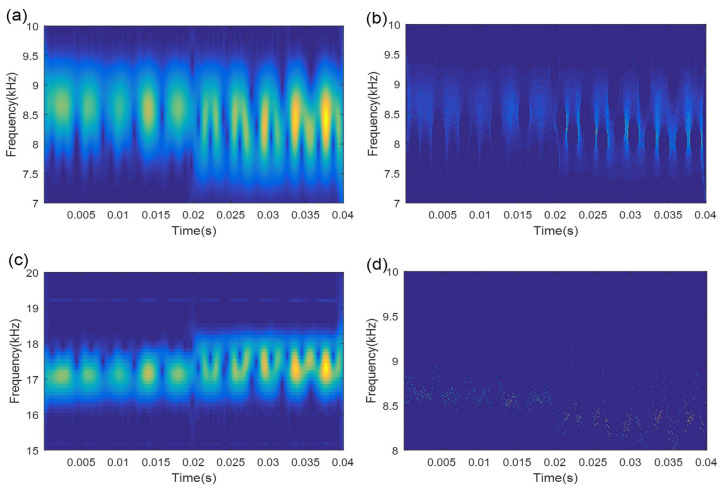
The analysis results of the compared methods for the inner race fault denoised signal: (**a**) STFT result; (**b**) MSST result; (**c**) WT result; (**d**) HHT result.

To fully verify the superiority of the proposed method, the fault signals after the SVD-based denoising are also processed by these four comparison methods. Figure 21 and Figure 22 show the results of these four comparison methods for analyzing the SVD-based denoising signals. Compared with analyzing the initial fault signals, the conditions of the TF energy concentration for the denoising signal using these four comparison methods are slightly better. However, the TFRs of the outer race fault and inner race fault remain ambiguous and cannot be identified.

## 7. Conclusions

In the actual working condition, the fault vibration signal of high-speed train axle bearing contains a lot of noise components. Although the TMSST method is able to produce clearer energy concentrated time−frequency (TF) trajectories to extract fault impulse features in the time−frequency representation (TFR), the interference of the background noise seriously restricts the TFR performance of the TMSST. To address this issue, a hybrid SVD-based denoising and self-adaptive TMSST is proposed to extract the fault feature of the axle bearing. On the one hand, a maximum SV mean method is put forward to determine the SV order to reconstruct the signal. The SVD-based denoising pre-processing technique can remove a large amount of background noise and reserve the useful fault impulse components. On the other hand, a minimum TFS-PEn evaluation criterion is presented to quantitatively evaluate the performance of TFR for the TMSST. Then, a water cycle algorithm is introduced into the TMSST to adaptively acquire the optimal algorithm parameters and to improve the reliability and robustness of the TMSST. Both the simulated fault signal analysis and experimental data prove the feasibility of the proposed method. In general, the proposed method fixes the shortcomings of the traditional TMSST and expands its application. Moreover, it has a certain value for axle bearing fault detection and provides a new way of thinking about the TFR of fault features, which is very likely to contribute to future related topics regarding other time−frequency analysis (TFA) techniques. The signal SVD and the optimization algorithm iteration process have a high computational cost. The main limitation of the proposed method is that it requires a large number of calculations and it is less efficient. Hence, improving the operating efficiency of the proposed approach still needs further research.

## Figures and Tables

**Table 1:** The geometric parameters of the axle bearing.

Type	Rolling Element Diameter	Pitch Diameter	Pitch Diameter	Contact Angle	Roller Number
FAG F-80781109	26.5 mm	185 mm	185 mm	10°	17

## Nomenclature

Time−frequency analysis	TFA
Time−frequency	TF
Spectral kurtosis	SK
Empirical wavelet transform	EWT
One-dimensional	1D
Two-dimensional	2D
Short-time Fourier transform	STFT
Wigner−Ville distribution	WVD
Wavelet transform	WT
Synchrosqueezing transformation	SST
Time-reassigned synchrosqueezing transformation	TSST
Time-reassigned multisynchrosqueezing transform	TMSST
Time−frequency representation	TFR
Singular value decomposition	SVD
Singular value	SV
Permutation entropy	PEn
Time−frequency spectrum permutation entropy	TFS-PEn
Water cycle algorithm	WCA
Group delay	GD
Hilbert-Huang transform	HHT
Time−frequency envelope spectrum	TFES
Time−frequency spectrum	TFS
